# The Doctrine of Probabilities in the Early Diagnosis of Peritonitis

**Published:** 1910-09

**Authors:** R. M. Harbin

**Affiliations:** Rome, Ga.


### Journal-Record of Medicine

**Successor to Atlanta Medical and Surgical Journal, Established 1855, and Southern Medical Record, Established 1870.**

**OWNED BY THE ATLANTA MEDICAL JOURNAL CO.**

**Published Monthly**

*Official Organ Fulton County Medical Society, State Examining Board, Presbyterian Hospital, Atlanta, Birmingham and Atlantic Railroad Surgeons' Association, Chattahoochee Valley Medical and Surgical Association, Etc.*

EDGAR G. BALLENGER., M. D., Editor.
BERNARD WOLFF, M. D., Supervising Editor.
A. W. STIRLING, M. D., C. M., D. P. H., J. S. HURT, B. Ph., M. D.
GEO. M. NILES, M. D., W. J. LOVE, M. D., (Ala.); Associate Editors.
E. W. ALLEN, Business Manager.

COLLABORATORS

Dr. W. F. WESTMORLAND, General Surgery.
F. W. McRAE, M. D., Abdominal Surgery.
H. F. HARRIS, M. D., Pathology and Bacteriology.
E. B. BLOCK, M. D., Diseases of the Nervous System.
MICHAEL HOKE, M. D., Orthopedic Surgery.
CYRUS W. STRICKLER, M. D., Legal Medicine and Medical Legislation.
E. C. DAVIS, A. B., M. D., Obstetrics.
E. G. JONES, A. B., M. D., Gynecology.
R. T. DORSEY, Jr., B. S. M. D., Medicine.
L. M. GAINES, A. B., M. D., Internal Medicine.
J. N. LeCONTE, M. D., Disease of the Stomach and Intestines.
L. B. CLARKE, M. D., Pediatrics.
EDGAR PAULIN, M. D., Opsonic Medicine.
THEODORE TOEPEL, M. D., Mechano Therapy.
R. R. DALY, M. D., Medical Society.
A. W. STIRLING, M. D., etc., Diseases of the Eye, Ear, Nose and Throat.
BERNARD WOLFF, M. D., Diseases of the Skin.
E. G. BALLENGER, M. D., Diseases of the Genito-Urinary Organs.

VOL. LVI. SEPTEMBER, 1910 NO. 6.

### THE DOCTRINE OF PROBABILITIES IN THE EARLY DIAGNOSIS OF PERITONITIS.

BY R. M. HARBIN, M. D., ROME, GA.

My apology for offering this discussion is that we should almost never lose a case of peritonitis if its incipiency could be promptly recognized, and oftentimes in the absence of positive symptoms we have to pursue the doctrine of probabilities, and we should endeavor to study this procedure until further efforts bring us more positive methods. While a diagnosis of the antecedent causes of a beginning peritonitis is of secondary importance, and is usually post-operative, a prompt recognition of a spreading peritoneal infection is of vital importance for the sake of surgical treatment, for peritonitis is a surgical disease, and in the light of post-operative diagnosis the so-called idiopathic type of the disease is a misnomer. Incidentally, it may be stated that the data upon which this discussion is based is derived from a study of 96 laporatomies for the causes of peritonitis. While some were hospital cases, the majority was seen in the homes, 76 having been seen in consultation from different communities, for any one of the smaller communities, or one general practice, however extensive, rarely furnishes enough cases to make a study of value.

While peritonitis is a disease for surgical treatment, the responsibility of early diagnosis frequently rests upon the medical attendant, who should lose no time in calling a consultant, if necessary, to establish a prompt diagnosis. The absorbent surface of the peritoneum is equal to that of the entire integument of the body, and a few hours of rapid absorption may determine irreparable damage. A surgical master in America has said that a good surgeon cannot always make a correct diagnosis before operation, but it is reprehensible in him not to do so after opening the abdomen. Of course there are exceptionally obscure cases in which to prolong an operation for that purpose might mean a sacrifice of the patient's remnant of vitality.

Increased experience usually makes the abdominal surgeon a more humble diagnostician in certain exceptional cases, and it is in this class of cases that we have to apply the doctrine of probabilities, for to wait for development of characteristic symptoms would be too late to make a diagnosis of greatest value, and under these circumstances it is just as important for the physician to be equipped with diagnostic skill as the surgeon, for he sees the cases first hand.

It is granted that operative treatment early in certain abdominal troubles may reveal the absence of suspected disorders. While the laity and some physicians are not educated to that point, it is only a question of time when early operations in cases of doubt will become established in the confidence of the laity. What proves to have been an unnecessary operation is at times looked upon by the uninformed as a crime, but time is fast dispelling such ignorance. Of course, that may happen in the hands of the best known surgeon, but the rarity of such an occurrence will be in direct proportion to the degree of diagnostic skill of the operator. So the doctrine of probabilities in one surgeon's hands will be different from that in another's.

Any criticism of the weakness of surgery applies with no less force to other branches of the profession, and we all are trying to better our methods.

In the 96 laporatomies that came under my observation, three proved a wrong diagnosis and two were negative, making a total error of 5.2 per cent. One was diagnosed abscess of the gall bladder, but a deep, inaccessible abscess of the liver ruptured three weeks later. Another was diagnosed rupture of the gall bladder, but no rupture was found. This will be referred to later in this discussion. The third case was believed to contain an interstitial pregnancy, as the left conuum of the uterus seemed to be sacculated. Before I saw her, the patient had had uterine hemorrhages for six weeks, for which she was curretted, and every few days the uterus was douched. The attending physician observed that the uterus was subjected to a ballooning process. She having a bloody, purulent discharge, was not examined per vagina the day of operation, and when the abdomen was opened a uniform and large distension of the uterus was seen. The ovaries were removed, and then a live foetus was curetted out of the left horn of the uterus. The foetus was fated because of a septic endometritis. The results were extremely satisfactory, for she had mental symptoms as well as toxæmic disturbances.

Dr. R. C. Cabot, of Boston (address, A. M. A., 1910,), in studying mistaken diagnosis from 1,000 autopsies, reviews 27 medical diseases in which correct diagnosis failed in 46 per cent. of the cases. Lobar pneumonia failed to be correctly diagnosed in 26 per cent., active phthisis 41 per cent., and typhoid fever 10 per cent. If clinical observers had the opportunities to verify diagnosis that surgery offers, these percentages would not be so high. Surgery may be called into the courts of the laity to account for its admitted weakness in early diagnosis, but as long as the practice of waiting for positive diagnosis obtains, the heavy mortalities will continue in evidence. The dangers from an unnecessary operation are nil in the hands of a well-equipped surgeon, and all objections are of the sentimental kind.

To be driven into operation or non-operation will usually bring heavy mortalities. Because positive early diagnosis is impossible, shall we allow many patients to lapse into incurable conditions? Statistics of the past stand out as a rebuke to such a policy. Probable diagnosis gets nearer the truth and saves more lives than negative diagnosis, for positive knowledge is only bought with the price of positive helplessness.

The established practice of early operation for appendicitis, after years of controversy, is firmly based on the doctrine of probabilities, in that we cannot predict the result of an individual case; and it only remains for the same rule of procedure to be applied to other abdominal lesions.

Gerster (Annals of Surgery, April, 1910,), in reviewing the causes of peritonitis, says: "Unhesitatingly it may be asserted that in the vast majority of cases, procrastination is more dangerous than operation," and any one who runs the course of peritonitis will read fatality.

In no region of the body is found greater difficulty in diagnosis, at times, than the abdomen, and it may not always be easy to recognize those cases which occupy the border line of doubt, for sometimes the apparently frank cases may have to be the exceptional ones. In fact, it is difficult to prognosticate the subsequent history of an individual case, for many of this class prove to have had transient disorders.

A common disorder arises from gastro-intestinal intoxication. While an alimentary fever occurs more frequently in children, it often occurs in adults as a confusing element in diagnosis. In every acute abdominal disorder, emptying the stomach and lower bowels and fasting, go a great way towards relieving toxic symptoms. A rising temperature under these circumstances should arouse grave suspicions, but it is more active than in a beginning peritonitis, and a lack of local rigidity and pain may create enough doubt to warrant waiting twelve to twenty-four hours, after which toxæmic symptoms rapidly vanish or else a characteristic colitis develops with mucous diarrhœa in which greater tenderness is observed on the left lower abdomen. A patient with an operation for the cure of hernia ran an active temperature for several days. When the lemon in the albumen was discontinued the fever subsided, only to be renewed when the lemon juice was resumed. Every other cause could be excluded and the wound remained aseptic.

The modern practice commands that every pain in the abdomen should receive careful study, however trivial it may seem to the patient, and the busy physician should let no duties interfere with frequent observation of abdominal cases during the first twenty-four hours. Under these circumstances the physician and surgeon should have consultation within the first thirty-six hours of abdominal disorders, for this is the golden opportunity for diagnosis as well as treatment. During this period of time the consultant must either make a diagnosis, or else decide that it would be safer to wait further for a positive conclusion. I know of no class of diseases where consultation is of greater value in maturing reliable opinions. Once by telling a patient to let me know next day if he was not better came near causing me to lose a case of perforative appendicitis. I had given such advice in a previous attack. Had I made more frequent visits I could have recognized the impending danger.

A young, stout nullipara whom I had consulted six months previous consulted me in reference to a bloody discharge from the uterus lasting six weeks along with cramp-like pains. Being suspicious, I urged her to keep me informed as to her condition. One week later she started to my office, but finally decided to call me. A vaginal examination revealed an ill-defined, boggy mass in the left pelvis. Two hours later the abdomen was opened at the hospital, and a ruptured tubal pregnancy was found, and the abdomen contained between a pint and a quart of fresh blood. My insistance in keeping up with the case enabled me to make an early diagnosis. I have often been convinced that patients living a distance from the physician have lapsed into incurable conditions for the reason that they have not been impressed with the importance of certain symptoms.

In estimating the probabilities, it is well to study the comparative frequency of the various causes of peritonitis. My own observation is limited to 96 laporatomies for the causes of peritonitis, as follows: Appendicitis 65, pelvic 13, tubal pregnancy 4, gall stone disease 5, strangulated hernia 2, wound of the kidney 1, traumatic rupture of the stomach 1, abscess of liver 2, fecal fistula 1, and unknown 2. Gerster (Annals of Surgery, April, 1910), in a review of 609 cases of peritonitis, noted the following causes: Appendicitis 461, gall stones 24, typhoid fever 23, trauma 17, strangulated hernia 16, gastric and duodenal ulcer 13, pelvic causes 12, volvulos and intussusception 8, pancreatic hemorrhage 6, liver abscess 3, thrombosis of mesentery 1, and unknown 20.

It has been estimated by other observers that 85 per cent. of the causes proceed from the appendix, and it has been assumed that 75 per cent of these cases of appendicitis undergo resolution from palliative treatment, perhaps to have subsequent attacks. By careful study some of the cases may be observed to improve in a few hours after emptying the stomach and lower bowels and fasting. It is very rare that a diagnosis of appendicitis will be difficult in the first twenty-four hours of the disease, and the abortive cases can usually be recognized. The patient should be seen every few hours during the day, and at the same time opiates should be withheld. If the symptoms in doubtful cases do not show signs of disappearance in twelve hours, operation should be advised. It has been accepted that operations in the first twenty-four hours are about as safe interval operations. About one per cent. of the cases undergo perforation, which usually brings about the initial symptoms, and this class of cases would perhaps give whatever mortalities that might follow twenty-four hour operations. The mortality directly depends on the duration that elapses from the time of escape of infection from the appendix and date of operation.

The remaining 24 per cent of cases proceed to the formation of abscess and secondary rupture and continued sepsis, producing heavy mortalities.

While the diagnosis of appendicitis is usually easy, a diagnosis of the different stages of the morbid process is difficult, if not impossible, and any reliable prognosis is manifestly untrustworthy. The probabilities should be explained to the patient, and if he is not willing to accept the advice of the surgeon, he should bear the burden of responsibility that arises from waiting. I have seen one case of the appendix on the left side, requiring a half hour's search to find it. The usual symptoms were present, however.

There are other disorders that are attended by epigastric pain and vomiting which may arise from acute indigestion with toxaema, a perforated gastric ulcer, gall stone colic or gastralgia. The latest word from extensive autopsies is that all grave abdominal disorders have an organic base. To wait for a confirmed diagnosis would be too late for a successful operation for perforated ulcer, and must be recognized within twenty-four hours. The probabilities, however, are this would be less frequent than any of the disorders giving similar symptoms, and the indications would require to be very strong in order to exclude all other disorders. Gastric ulcer, untreated surgically, gives a fatality of 25 per cent., and six per cent of the cases undergo perforation, while eight per cent. have hemorrhage. (Johnson's Surgical Diagnosis, Vol. II, page 40.) The signs are those of shock and hemorrhage. According to the Mayo brothers, out of 27 cases of ulcer 11 were found in the stomach and 16 in the duodenum.

Intestinal obstruction furnishes another abdominal crisis. Gibson observed 1,000 operations for this trouble, of which 35 per cent. arose from strangulated hernia, 19 per cent. from intussusception and 12 per cent. from volvulus.

There are certain cases of peritonitis in the aged that give almost a normal course of pulse and temperature throughout. Recently a man seventy years old had an acute abdominal pain. He took calomel and castor oil which produced several evacuations. I saw him twelve hours later and found normal pulse and temperature, with an indurated and elongated mass in the region of the ascending colon with much tympany. Attempts to get further evacuations were futile, and operative interference was declined. After washing out the stomach and fasting for two days, with no sign of toxæmia, the bowels acted profusely, with partial disappearance of the mass, but symptoms of grave sepsis and general peritonitis repidly developed and he died in thirty-six hours. Evidently a circumscribed abscess from a perforated appendix produced intestinal obstruction by contiguous adhesions, yet there were none of the usual signs of sepsis.

I recall the case of a woman 35 years old having incessant vomiting, temperature 102 F., pulse 160, and extreme tympany, in which the only clue to the cause was a sudden disappearance of a mass in the region of the gall bladder observed by the attending physician. A rapid operation for drainage was accomplished in eight minutes, which brought away bile and pus. She recovered. Another case of a woman of same age and symptoms with jaundice, having been treated for several years for gall stones, with a pulse of 180 and temperature 102, revealed at operation only adhesions of the hepatic flexure of the colon omentum and a portion of the greater curvature of the stomach to a shrunken gall bladder. The attending physicians had observed what they believed to be a sudden disappearance of a tumor about the gall bladder. The liver markedly bulged downward, perhaps due to a deep abscess. Her condition and environments did not warrant further manipulation, and she died four days later.

The most important and frequent lesions occur in the right abdomen, and pain in this region should lead us to suspect fecal impaction, colitis with adhesions, appendicitis, prolapsed and incarcerated kidney, nephro-lithiasis, perinephric abscess, pyonephrosis, salpingitis and extra-uterine pregnancy.

A purge usually eliminates the diagnosis of impaction. Once I was requested to go to a nearby town to operate for abscess of the appendix. Not believing it to be a case of impaction, we decided as a means of exclusion to give two ounces of castor oil and wait five hours on the result, which was disappearance of the mass; yet other seemingly effective purgatives had been given. Still another case was seen where a mass the size of a lemon could be felt after quite thorough purging. A diagnosis of appendical abscess was made, but operation was declined, and the next day a bolus of cheese was discharged.

As a rule, if pain, tenderness, tumor and fever persist after purging, the disease is of a recent, inflammatory nature, if the pre-existence of tumor can be excluded. Colitis requires to be excluded, which can usually be done by bearing in mind a history of previous attacks, direct imprudence in eating, diarrhœa and mucous stool. It is the rare exception to have diarrhœa in a spreading peritonitis. An enlarged and prolapsed kidney may be mistaken for appendical abscess, especially if there exist signs of inflammatory adhesions. The symptoms, however, are less severe, and the presence or absence of the kidney from its normal habitat may be detected.

An enlarged suppurating kidney can usually be recognized by a careful urinalysis and tumor. In every case of chronic appendicitis a careful urinalysis should be made. The appendix shows frequent association with ovary in morbid processes. A few months ago a case came under observation that gave a history of attacks of appendicitis with increased pain during menstruation. Operation revealed the presence of adhesions between the mesentery of the appendix and ovary.

Salpingitis usually gives pain lower in the abdomen, and is not so intimately associated with intestinal disorders, and the mistake should never be made of operating in these acute cases. I once made the mistake of diagnosing a case as convalescing appendicitis, when later an extra-uterine pregnancy was found. Another case of extra-uterine pregnancy was given an alternate diagnosis of pelvic abscess. After puncturing the sac by vaginal section, an alarming hemorrhage occurred in the abdomen from rupture of the sac, and required the abdominal operation to arrest it. A fluctuating tumor in the pelvis, showing irregular growth, should arouse suspicion of extra-uterine pregnancy.

### Summary.

1. Peritonitis is a surgical disease, and the so-called idiopathic variety is a misnomer.

2. There should be no fatalities if the diagnosis could be made early.

3. The medical attendant should advise consultation within twenty-four hours of the disease, for no other field of diagnosis is more fruitful of good from frequent consultations.

4. In the absence of positive symptoms the doctrine of probabilities should be pursued, which is now the accepted rule of procedure in appendicitis.

5. In 96 laporatomies for the causes of peritonitis there was an error of 5.2 per cent. in diagnosis.

6. Delay in first forty hours is more dangerous than operation, for positive diagnosis may be bought with the price of positive helplessness.

7. Every acute abdominal trouble should receive frequent observations and careful study until the diagnosis is made, or else arrive at a decision as to the safety of waiting.

8. Seventy-four per cent. of the causes of peritonitis proceed from the appendix.

9. The responsibility of delay of operation for appendicitis should be borne by the patient.

10. Extensive autopsies prove that all serious abdominal disorders have an organic base.

11. Peritonitis in the aged usually gives at first normal temperature and pulse curves.

12. Acute alimentary disturbances are to be thought of as more common in children than peritoneal infections.

13. The right abdomen furnishes the more common foci for infections.

14. Colitis is one of the most common factors in confusing an early diagnosis of peritonitis.

